# Microstructural, Mechanical, and Thermal Properties of Textured Si_3_N_4_/BN Composite Ceramics Prepared Using Two-Step Sintering

**DOI:** 10.3390/ma18153573

**Published:** 2025-07-30

**Authors:** Dexiang Gong, Yi Zhou, Yunwei Shi, Qianglong He

**Affiliations:** 1 School of Materials Science and Engineering, Wuhan University of Technology, Wuhan 430070, China; gongdexiang2025@163.com (D.G.); yizhou02gg@163.com (Y.Z.); 917069803@whut.edu.cn (Y.S.); 2 Hubei Longzhong Laboratory, Xiangyang 441000, China

**Keywords:** Si_3_N_4_/BN composite ceramics, texture, PHIP process, mechanical properties, thermal conductivity

## Abstract

Textured Si_3_N_4_/BN composite ceramics were successfully fabricated using two-step sintering, combining pseudo-hot isostatic pressing (PHIP) and gas pressure sintering. The grain size of h-BN platelets had a significant influence on densification and mechanical and thermal properties. With an increase in h-BN grain size, the volume density of the composite ceramics gradually decreased, while flexural strength gradually increased. Meanwhile, larger h-BN platelets were more likely to trigger toughening mechanisms like large-angle deflection and greatly increase fracture toughness. Through proper selection of h-BN grain size, textured ceramics, with the addition of h-BN platelets of 1–2 μm, showed high thermal conductivity (∼92 W∙m^−1^∙K^−1^) and reliable mechanical properties (∼540 MPa, ∼7.5 MPa∙m^1/2^, ∼11.1 GPa). Therefore, texture control is an effective means of improving the overall performance of ceramic materials. Novel textured composite ceramics thus have great potential in large-scale fabrication and directional heat dissipation applications.

## 1. Introduction

Due to their superior mechanical properties, good thermal conductivities, electrical insulation effects, and similar thermal expansion coefficients to semiconductor devices, Si_3_N_4_ ceramics hold potential as ceramic substrate materials for the heat dissipation of highly integrated and high-power-density chips [1,2,3,4,5,6]. Nevertheless, the thermal conductivity of monothetic Si_3_N_4_ ceramics is highly sensitive to lattice oxygen content, grain-boundary thickness, and grain size [7]. During sintering, oxygen atoms quickly dissolve into Si_3_N_4_ crystals and form vacancies of lattice oxygen, leading to phonon scattering and impairing thermal conductivity. Generally, researchers carefully select additives and sintering procedures to minimize Si_3_N_4_ content [8,9,10,11]. Through simple doping of an extra 5 mol% of carbon to clean grain boundaries and reduce oxygen content, Si_3_N_4_ ceramics achieve a significant improvement of ∼25.5% in thermal conductivity from 102 to 128 W∙m^−1^∙K^−1^ under the same sintering conditions [9]. Meanwhile, high sintering temperatures and prolonged dwelling times are used to accelerate grain growth and form a thin glassy phase at the grain boundary. For example, Li et al. [12] prepared high-thermal-conductivity Si_3_N_4_ ceramics (132 W∙m^−1^∙K^−1^) using gas pressure sintering at 1900 °C for 12 h. However, high sintering temperatures and long dwelling times result in higher preparation costs, thus restricting the further industrial application of such ceramics.

Hexagonal boron nitride (h-BN) platelets also exhibit electrical insulation effects and an excellent in-plane thermal conductivity of nearly 400 W∙m^−1^∙K^−1^ [13], with the simulated *c*-axis thermal conductivity of individual β-Si_3_N_4_ crystals reaching ∼450 W∙m^−1^∙K^−1^ [14]. According to a previous study of ours [15], h-BN platelets and β-Si_3_N_4_ rod-like grains tend to rotate toward sintered planes during the axial compression induced by hot pressing, and the texture degree increases with the compressive strain. This synergetic effect of h-BN and β-Si_3_N_4_ grains along the sintered plane represents a feasible approach to achieving a significant enhancement of thermal properties. Most studies of Si_3_N_4_/BN composite ceramics concentrate on densification, machinability, and mechanical, dielectric, and thermal shock resistance properties [16,17,18,19,20,21,22]. They rarely explore the possibility of using h-BN additives to optimize the thermal conductivity of composite ceramics.

In this study, textured Si_3_N_4_/BN composite ceramics were fabricated via two-step sintering, combining pseudo-hot isostatic pressing (the PHIP process) with gas pressure sintering (GPS). Compared with SPS techniques [23,24,25], the combination of PHIP and GPS more effectively enabled the large-scale preparation of reliable ceramic materials. The h-BN and β-Si_3_N_4_ grains oriented along the sintered plane greatly optimized the thermal conductivity of composite ceramics, compelling us to further analyze this synergetic effect. Furthermore, the effects of h-BN grain size on densification, mechanical properties, and thermal conductivity were investigated in detail.

## 2. Experimental Procedures

**Figure 1:**
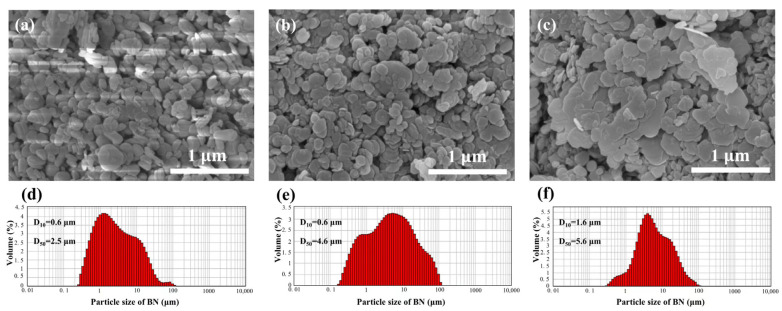
The grain morphology and size distribution of the raw h-BN powders. (**a**,**d**): nm; (**b**,**e**): 1–2 μm; (**c**,**f**): 5–10 μm.

**Figure 2:**
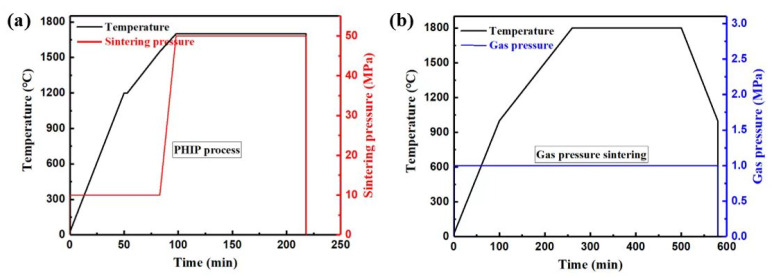
Schematic illustration of the two-step sintering: (**a**) PHIP process, (**b**) Gas pressure sintering.

Commercial α-Si_3_N_4_ powders (α phase >95%, 250 nm, UBE Industries., Ltd., Yamaguchi, Japan) were used during the main phase (80 vol%), while h-BN powders (purity >99.9%, Shanghai Maclin Biochemical Technology Co., Ltd., Shanghai, China) were used during the second phase (20 vol%). A 6 wt% Mg_2_Si powder (purity >99.5%, 10 μm, Zhejiang Yamei Nano Technology Co., Ltd., Wenzhou, China) and 2 wt% Y_2_O_3_ (purity >99.99%, 50 nm, Shanghai Aladdin Biochemical Technology Co., Ltd., Shanghai, China) were used as additives. Reagent bottles were labeled with three h-BN grain sizes: nm, 1–2 μm, and 5–10 μm. The initial grain size distribution of the raw h-BN powders was determined using a particle size analyzer (Mastersizer 2000, Malvern Panalytical, Malvern, Worcestershire, UK) and is shown in Figure 1, alongside the grain morphology. The initial particle sizes (D50) were 2.5, 4.6, and 5.6 μm. The Si_3_N_4_/BN composite ceramic samples were labeled as SNBN1, SNBN2, and SNBN3. The powder mixture was then ball-mixed in a highly pure ethanol medium containing SiO_2_ balls for 12 h. A rotary evaporator was used at 65 °C to remove residual ethanol in the slurry, and then the mixed powders were dried further at 65 °C for 24 h in a vacuum oven. Finally, they were passed through a 200-mesh screen to obtain well-dispersed powders. The samples were prepared by means of two-step sintering, combining pseudo-hot isostatic pressing (the PHIP process) and gas pressure sintering, as shown in Figure 2. The sintering procedure was identical to that in a previous study [26]. Before the PHIP process, pre-sintered samples with a diameter of 36 mm were prepared at 1550 °C for 30 min under a sintering pressure of 15 MPa. During the PHIP process, the pre-sintered samples were placed in a graphite die center and then embedded in the mixed Si_3_N_4_/BN powders (1:1). The samples were subsequently sintered in a hot-pressing furnace (916G-G Press, Thermal Technology Inc., Santa Rosa, CA, USA) at 1700 °C for 2 h, followed by further sintering in a gas pressure sintering furnace (FPW 180/250-2-2200, FCT Anlagenbau GmbH, Rauen, Germany) at 1800 °C for 4 h. The nitrogen pressure was set to 1 MPa. After the sintering procedure, all samples were naturally cooled to room temperature.

The volume density was measured using Archimedes’ method. X-ray diffraction (XRD, PANalytical Empyrean, Almelo, The Netherlands) patterns were detected to determine the phase compositions. A field-emission scanning electron microscope (FESEM, SU8230, Hitachi, Tokyo, Japan) and transmission electron microscope (TEM, Talos F200S, Hillsboro, OR, USA) were simultaneously utilized to observe the fresh fracture surfaces and high-resolution phase distribution of Si_3_N_4_/BN composite ceramics. To determine the effect of microstructural anisotropy on mechanical and thermal properties in different directions, testing directions along different planes were defined, namely, the D1 and D2 testing directions, parallel and perpendicular to the direction of sintering pressure, respectively. Before Vickers hardness was measured, the surfaces were polished with a polishing machine (Alpha-600, Trojan (Suzhou) Material Technology Co., Ltd., Suzhou, China) to enhance the mirroring effect. During Vickers hardness testing (FALCON 400, INNOVATEST Shanghai Co., Ltd., Shanghai, China), the load and the dwell time were 1 kg and 15 s, respectively. The standard bars of flexural strength and fracture toughness tested by the ceramics testing system (CMT6503, Ji’nan Meitesi Testing Technology Co., Ltd., Jinan, China) measured 3 × 4 × 36 and 2.5 × 5 × 25 mm^3^, respectively. During the three-point bending test, the loading rates were 0.5 and 0.05 mm/min and the spans were 30 and 20 mm, respectively. The fracture toughness was evaluated by means of the single-edge notched beam method with a notch of <0.1 mm using the following formulas [27]:

(1) $$K_{IC}=\;\frac{3FS_{0}\alpha^{\frac{1}{2}}}{2BW^{\frac{3}{2}}{\left(1-\alpha \right)}^{\frac{3}{2}}}Y$$

(2) $$Y=1.9887-1.326\alpha -(3.49-0.68\alpha +1.35\alpha^{2})\alpha (1-\alpha ){\left(1+\alpha \right)}^{-2}$$

where *F* (N) is the load limit; *S*_0_ (mm) is the span distance; *W* (mm) is the bar height; *a* (mm) is the notch depth (0.4 < *a*/*W* < 0.6); *B* (mm) is the bar width; *α* is the *a*/*W* ratio; and *Y* is the stress intensity shape factor. Each testing result is the average of six values. After flexural strength testing, fracture roughness was determined using the VHX digital microscope (KEYENCE VHX-7000, KEYENCE Corporation, Osaka, Japan). The thermal conductivities at room temperature were obtained by means of a laser flash apparatus (LFA457, Netzsch, Selb, Germany). The samples were then processed to a size of 6 × 6 × 1.8 mm^3^. According to the specific heat of monothetic h-BN (0.85 J∙g^−1^∙K^−1^) and β-Si_3_N_4_ (0.68 J∙g^−1^∙K^−1^), that of the composite ceramics was calculated to be 0.71 J∙g^−1^∙K^−1^. Meanwhile, the thermal conductivity was calculated using a previously established formula [28]. Three samples (2 × 3 × 18 mm^3^) of each composite ceramic were prepared to obtain the stress–strain curves under different loading rates. The testing span was set to 15 mm, and the loading rates were 0.05, 0.1, and 0.5 mm/min, respectively.

## 3. Discussion

### 3.1. Phase Analysis

**Figure 3:**
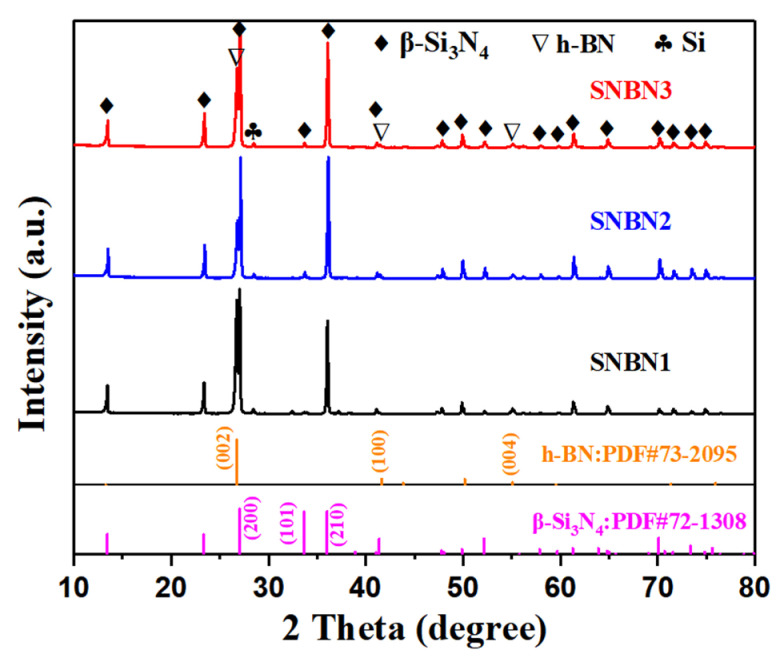
XRD patterns of Si_3_N_4_/BN composite ceramics on sintered planes.

The phase compositions of the composite ceramics on sintered planes are shown in Figure 3. The XRD patterns demonstrate the existence of β-Si_3_N_4_, h-BN, and Si, while the Mg_2_Si and Y_2_O_3_ additives are not detected. The presence of Si is possibly attributable to reactions (3–5) [29,30]. During sintering, the thermal decomposition reaction of the Mg_2_Si additive occurs around 1100 °C [2,29,31]. The generated Mg may further react with SiO_2_ on the surface of Si_3_N_4_ powders, thereby producing a Si phase. Moreover, the Mg_2_Si additive may directly react with N_2_ under a N_2_ atmosphere to produce Si. However, this by-product of the Mg element is not detected in the XRD patterns. With a rise in sintering temperature, the by-product would react with Y_2_O_3_ to form an amorphous eutectic phase, and then the liquid sintering mechanism would occur. The Mg element is mostly distributed in the grain-boundary glassy phase, making it undetectable. The generated Si would induce silicothermic reduction to decrease the oxygen content and increase the N/O ratio in a liquid phase, which is beneficial to the nucleation and grain growth of β-Si_3_N_4_ [11]. Compared with the standard X-ray diffraction peaks of β-Si_3_N_4_ and h-BN, the (*hk*0) peaks of the β-Si_3_N_4_ phase evidently become stronger, while the (*hkl*) peaks almost disappear. Meanwhile, the h-BN platelets show a similar phenomenon: the (002) and (004) peaks become the main peaks, while the (100) peaks almost disappear. This indicates that the rod-like β-Si_3_N_4_ grains and h-BN platelets exhibit preferential orientation and tend to distribute along sintered planes. This bimodal texture can be attributed to the large compressive strain induced by the PHIP process, as large deformation is favorable for anisotropic grain rotation.

Mg_2_Si → 2Mg(g) + Si (3)

2Mg(g) + SiO_2_ → 2MgO + Si (4)

3Mg_2_Si + 2N_2_(g) → 2Mg_3_N_2_(amorphous) + 3Si (5)

**Figure 4:**
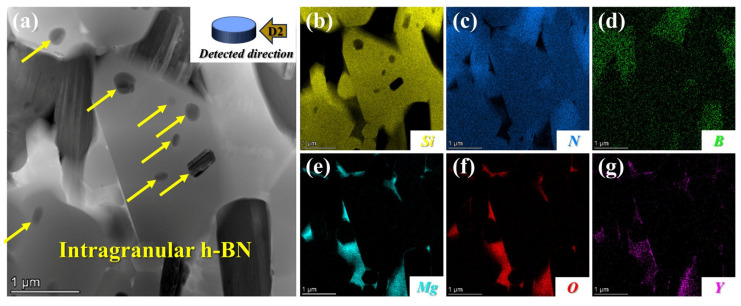
(**a**) Bright-field TEM image of SNBN3 (Yellow arrows indicate Intragranular h-BN); (**b**–**g**) EDS elemental distribution maps of Si, N, B, Mg, O, and Y. Detected direction is along D2.

High-resolution TEM and EDS mapping micrographs of SNBN3 are shown in Figure 4. It is evident that different h-BN platelet morphologies are associated with different distributions. Nano-sized h-BN particles, originating from nano h-BN (<500 nm) in the raw h-BN powders, are dispersed within large β-Si_3_N_4_ grains and form an intragranular h-BN phase, as demonstrated in Figure 1. However, micro-sized h-BN platelets are mainly observed in an intergranular phase located at the grain boundary. During anisotropic growth of β-Si_3_N_4_, the grain boundary inevitably comes into contact with h-BN grains. Because less boundary energy is consumed, the grain boundary of β-Si_3_N_4_ can easily overlap with the nano-sized h-BN (<500 nm) [32]. However, when the h-BN grain size is large, the grain boundary only moves along the h-BN boundary. The variation in distribution with h-BN grain morphology is also proven by the elemental distribution maps in Figure 4b–d. Maps for the Mg, O, and Y elements, mainly distributed in the grain-boundary glassy phase, are shown in Figure 4e–g. These results are in accordance with the absence of magnesium compounds in the XRD patterns above.

### 3.2. Microstructural Characterization

**Figure 5:**
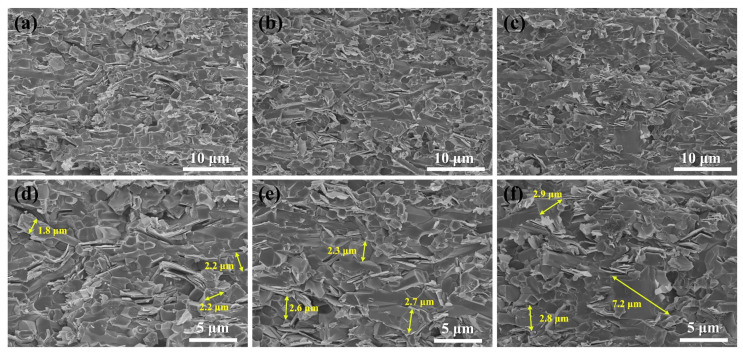
FESEM images of the fracture surfaces parallel to the cross-section of samples with different h-BN grain sizes: (**a**,**d**) SNBN1; (**b**,**e**) SNBN2; (**c**,**f**) SNBN3.

The fresh fracture surfaces created after flexural strength testing, parallel to the cross-sections of SNBN1, SNBN2, and SNBN3, are shown in Figure 5. No pores are observed on these surfaces. The fracture mode of the Si_3_N_4_ grains is predominantly typical transgranular fracture. Most of these rod-like β-Si_3_N_4_ grains show a hexagonal fracture morphology on the fracture surfaces, with some of the hexagonal prism exposed. On the other hand, platelets simultaneously appear in transgranular fracture and pull-out modes. Lamellar pits and prominences can be clearly observed on the fracture surfaces. This can be attributed to the difference between the internal and external bonding strength of the h-BN crystals. In an individual h-BN grain, the multiple-layer structure is only bonded by weak van der Waals forces. However, at the interface between the h-BN and grain-boundary phases, many covalent bonds exist. When crack tips pass through h-BN grains, transgranular fracture first occurs at the interface, and then weak van der Waals forces between layers induce crack propagation along the layer until a limit is reached. Eventually, the crack tips once again transgranularly propagate through the h-BN grains. Rod-like grains and platelets thus demonstrate preferential orientation and distribution along the sintered planes, yielding a bimodal texture.

The addition of a second phase would lead to steric hindrance and influence the growth of β-Si_3_N_4_. SNBN1, SNBN2, and SNBN3 all contain the same volume of h-BN platelets. As shown in Figure 5d–f, with the increase in h-BN platelet size, the grain diameter of β-Si_3_N_4_ gradually increases, with the largest β-Si_3_N_4_ grain diameters in SNBN1, SNBN2, and SNBN3 being 2.2, 2.7, and 7.2 μm, respectively. The finer h-BN grains in SNBN1 are well distributed, which greatly hinders the growth of β-Si_3_N_4_ grains. This difference in microstructure could influence the fracture behavior and mechanical and thermal properties.

### 3.3. Anisotropy of Mechanical and Thermal Properties

**Figure 6:**
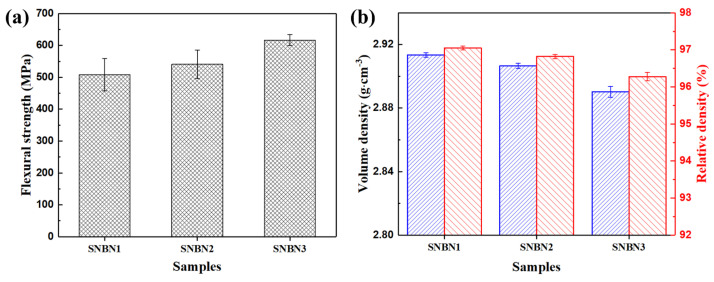
Flexural strength and density of samples with different h-BN grain sizes: (**a**) flexural strength; (**b**) volume density and relative density.

Figure 6 shows the flexural strength and volume density of SNBN1, SNBN2, and SNBN3. With the increase in h-BN grain size, the measured flexural strength values become 507.60 ± 50.67, 540.60 ± 44.47, and 616.68 ± 16.91 MPa, respectively, as demonstrated in Figure 6a. The volume density values in Figure 6b exhibit a reverse tendency, being 2.913 ± 0.001, 2.907 ± 0.002, and 2.89 ± 0.003 g/cm^3^, respectively. The relative densities of all samples are approximately 97%, which is relatively high [33,34]. Generally, the addition of finer h-BN grains is effective in obtaining more-densified ceramics. Larger h-BN grains would form a “card” structure, impairing the densification process during sintering. The results indicate that SNBN1, with its higher volume density and finer h-BN grains, exhibits the lowest flexural strength. SNBN3 has larger rod-like β-Si_3_N_4_ grains, which would consume more fracture energy due to the stronger self-toughening mechanism on sintered planes. This demonstrates that larger h-BN grains are more effective in improving the mechanical properties of Si_3_N_4_/BN composite ceramics. Moreover, the addition of finer h-BN platelets may result in the increased formation of the intragranular h-BN phase in composite ceramics, representing a structural flaw, as this would degrade the mechanical properties.

**Figure 7:**
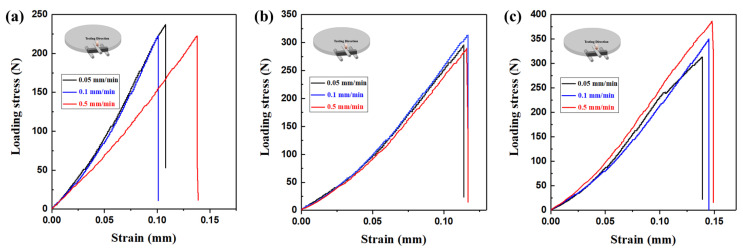
Stress-strain curves of Si3N4/BN composite ceramics with different h-BN grain size under different loading rates: (**a**) SNBN1 sample, (**b**) SNBN2 sample, (**c**) SNBN3 sample.

Three samples of each composite ceramic were tested to obtain the stress–strain curves under different loading rates. All samples display classical brittle fracture during flexural strength testing. In Figure 7, it is evident that the strain values tend to increase with the loading rate. Moreover, with the increase in h-BN grain size of the composite ceramics, the stress values show a steady increase when loading stress reaches its limit. Therefore, h-BN grain size has a significant influence on the flexural strength of Si_3_N_4_/BN composite ceramics.

**Figure 8:**
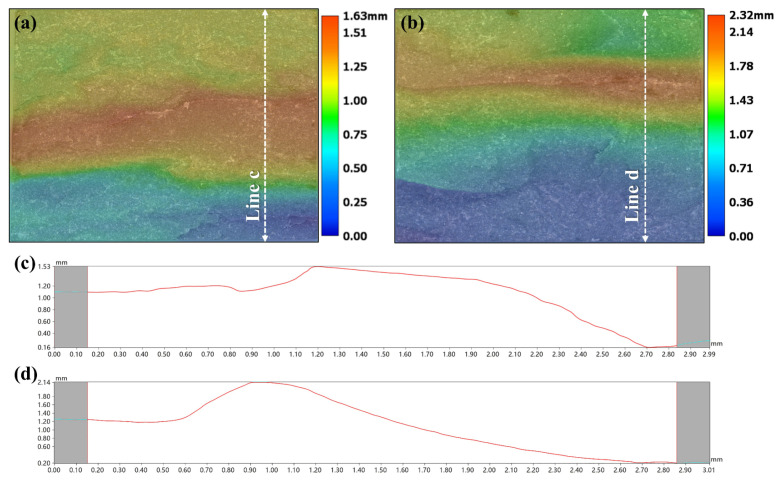
Surface morphology and line scan of fracture roughness after flexural strength testing of samples: (**a**,**c**) SNBN1; (**b**,**d**) SNBN3.

The surface morphology of fracture roughness of SNBN1 and SNBN3 after flexural strength testing is shown in Figure 8. When fine h-BN grains are added to composite ceramics, the distance between the top and bottom is 1.63 mm. When the h-BN grain size increases to 5–10 μm, this distance increases to 2.32 mm, and the sample displays rougher fracture. To further investigate roughness on the fracture surfaces, line scans in the vertical direction are employed. The fracture surface of SNBN1 appears to be Z-shaped. It shares this characteristic with the two-dimensional textured Si_3_N_4_ ceramics. The fracture would propagate in the direction of grain orientation until the sample reaches its load limit. The distance between the initial fracture platform and the top is about 0.4 mm. On the other hand, the fracture surface of SNBN3 is V-shaped, and the distance between the initial fracture platform and the top increases to about 0.9 mm. This indicates that SNBN3 would absorb more fracture energy before reaching fracture failure, yielding a rougher fracture surface. Therefore, the increase in h-BN platelet size is effective in deflecting crack propagation and increasing the fracture resistance of Si_3_N_4_/BN composite ceramics.

**Figure 9:**
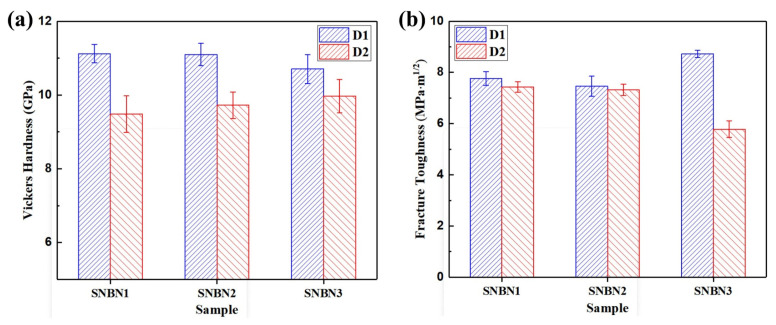
(**a**) Vickers hardness and (**b**) fracture toughness of Si_3_N_4_/BN composite ceramics with different h-BN platelet sizes in different directions.

The bimodal texture along the sintered plane would affect the mechanical properties in different directions and lead to unique anisotropy. The Vickers hardness and fracture toughness of Si_3_N_4_/BN composite ceramics in different directions are shown in Figure 9. The D1 Vickers hardness (Hv1) values of SNBN1, SNBN2, and SNBN3 are 11.12 ± 0.25, 11.10 ± 0.31, and 10.71 ± 0.39 GPa, respectively, while those in the D2 direction are 9.48 ± 0.50, 9.73 ± 0.36, and 9.97 ± 0.45 GPa. Thus, with the increase in h-BN platelet size, Vickers hardness gradually decreases along D1 and increases along D2. In SNBN3, the anisotropy of Vickers hardness rarely disappears. The matrix of the Si_3_N_4_/BN composite ceramics comprises the rod-like β-Si_3_N_4_ phase, with the two-dimensional distribution of these grains along sintered planes leading to Vickers hardness anisotropy. The sintered planes would exhibit a higher Vickers hardness. Generally, the addition of h-BN softens Si_3_N_4_/BN composite ceramics [19]. With the increase in the h-BN grain size, increased formation of intergranular h-BN platelets would decrease hardness on the sintered plane. This would lead to a decrease in the anisotropy of Vickers hardness of the textured Si_3_N_4_/BN composite ceramics. The D1 fracture toughness values of SNBN1, SNBN2, and SNBN3 are 7.76 ± 0.26, 7.46 ± 0.40, and 8.72 ± 0.14 MPa∙m^1/2^, respectively, while those in the D2 are 7.43 ± 0.20, 7.32 ± 0.22, and 5.78 ± 0.33 MPa∙m^1/2^. The fracture toughness of SNBN1 and SNBN2 along different directions is thus similar. SNBN3 shows high fracture toughness anisotropy, reaching 51%. Compared with SNBN1 and SNBN2, the D1 fracture toughness of SNBN3 is improved by 12.37% and 16.89%, respectively. This indicates that tiny h-BN platelets are unable to trigger the toughening mechanism. In contrast, increased formation of the intragranular h-BN phase would degrade microstructure reliability and properties. However, the tiny h-BN platelets are favorable for the densification process of Si_3_N_4_/BN composite ceramics, which would improve the overall Vickers hardness of ceramics to some degree.

**Figure 10:**
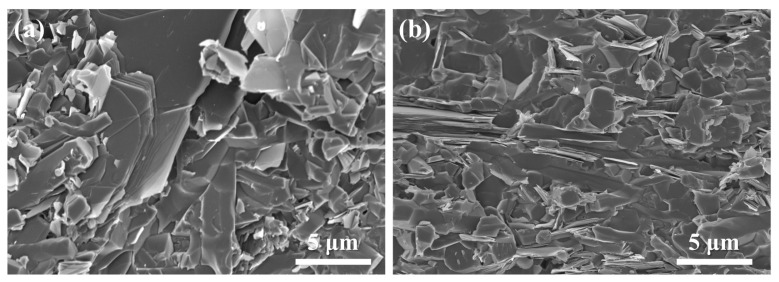
Fracture surfaces of SNBN3 after testing in different directions: (**a**) D1; (**b**) D2.

The fracture surfaces of SNBN3 after fracture toughness testing in different directions are shown in Figure 10. The fracture modes in the different testing directions are completely different. As shown in Figure 10a, many long, rod-like β-Si_3_N_4_ grains and lamellar h-BN grains are exposed on the fracture surface. The fracture mode of SNBN3 along D1 is dominated by intergranular fracture. The presence of a multiple-layer h-BN structure reveals that large h-BN platelets effectively deflect the crack tips along the layers to increase the fracture energy consumed and fracture resistance. This is possible due to the bimodal texture of the Si_3_N_4_/BN composite ceramics along the sintered plane. Nevertheless, the random grain orientation in the D2 direction somewhat affects the crack propagation direction, making it difficult to improve the fracture toughness. Therefore, the fracture mode of h-BN and β-Si_3_N_4_ along D2 is dominated by transgranular fracture, with a few h-BN platelets demonstrating pull-out phenomena.

**Figure 11:**
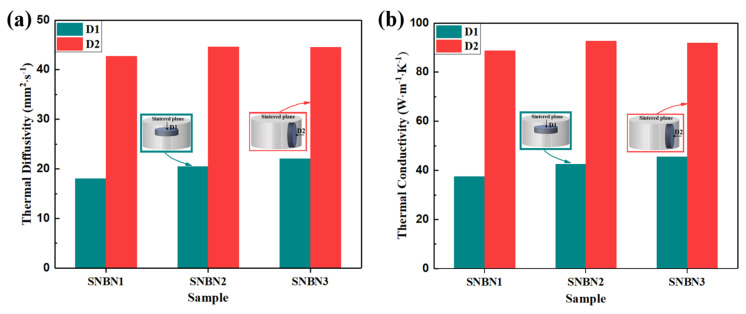
(**a**) Thermal diffusivity and (**b**) thermal conductivity between room temperature and 400 °C of Si_3_N_4_/BN composite ceramics with different h-BN platelet sizes in different directions.

Figure 11 displays the thermal diffusivity and thermal conductivity of textured Si_3_N_4_/BN composite ceramics with different h-BN platelet sizes in different directions. The h-BN platelets and rod-like β-Si_3_N_4_ grains are oriented along the sintered plane. The individual h-BN platelets exhibit high in-plane thermal conductivity, while the individual rod-like β-Si_3_N_4_ grains display high *c*-axis thermal conductivity. This provides a potential opportunity to enhance the thermal conductivity along the sintered plane. As shown in Figure 11a, the thermal diffusivity values along D1 obtained using the laser flash apparatus are 18.04, 20.52, and 22.05 mm^2^∙s^−1^ in SNBN1, SNBN2, and SNBN3, respectively, while those along D2 are 42.67, 44.62, and 44.53 mm^2^∙s^−1^. As shown in Figure 11b, the thermal conductivity values of the Si_3_N_4_/BN composite ceramics along D1 are 37.52, 42.59, and 45.49 W∙m^−1^∙K^−1^, respectively, while those along D2 are 88.75, 92.61, and 91.88 W∙m^−1^∙K^−1^. Under a low sintering temperature of 1800 °C and short dwelling time of 4 h, all textured Si_3_N_4_/BN composite ceramics achieve high thermal conductivity (∼90 W∙m^−1^∙K^−1^).

**Figure 12:**
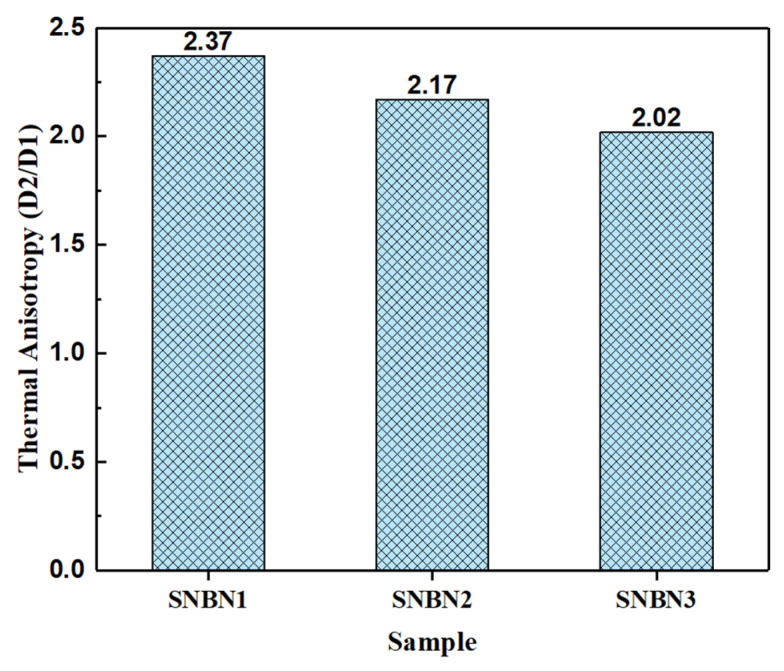
Thermal anisotropy of Si_3_N_4_/BN composite ceramics with different h-BN platelet sizes.

**Figure 13:**
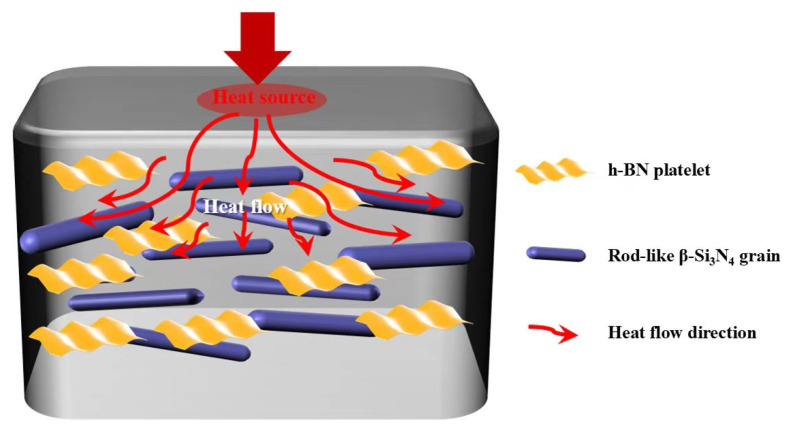
Schematic diagram of anisotropic thermal transfer induced by grain orientation of textured Si_3_N_4_/BN composite ceramics.

Thermal anisotropy is calculated using the D2/D1 ratio. The thermal anisotropies of the Si_3_N_4_/BN composite ceramics are shown in Figure 12. The values gradually decrease with the increase in h-BN grain size, being 2.37, 2.17, and 2.02, respectively. This reveals that the addition of tiny h-BN grains is effective in enhancing the anisotropy of grain orientation and forming a highly textured microstructure. The mechanism of thermal conductivity enhancement induced by such a microstructure is shown in Figure 13.

A comparison of the properties of the textured Si_3_N_4_/BN composite ceramics with those of other Si_3_N_4_- and/or BN-based ceramics is shown in Table 1. The h-BN-based composite ceramics generally show high thermal conductivity (>90 W∙m^−1^∙K^−1^) and non-ideal mechanical properties (<300 MPa, <3 MPa∙m^1/2^) [35,36]. Meanwhile, under a low sintering temperature and short dwelling time, the Si_3_N_4_ ceramics show the opposite tendency, with low thermal conductivity (<80 W∙m^−1^∙K^−1^) and favorable mechanical properties (>800 MPa, >7 MPa∙m^1/2^) [7,8]. Nevertheless, it is difficult to create a composite of Si_3_N_4_ and BN phases that combines the benefits of both [33]. In this study, SNBN2 prepared by means of two-step sintering showed more reliable overall performance than previously reported products. Moreover, different grain sizes of raw h-BN powders could be selected to create Si_3_N_4_/BN composite ceramics with various properties, thereby satisfying diverse application needs.

## 4. Conclusions

In this study, textured Si_3_N_4_/BN composite ceramics were successfully fabricated using two-step sintering, combining the PHIP process and gas pressure sintering. The effects of h-BN grain size on the densification process, texture degree, and mechanical and thermal properties were studied in detail. The following conclusions were drawn:

(1) The h-BN platelets and rod-like β-Si_3_N_4_ grains are distributed on the sintered plane in an orderly fashion. This textured microstructure yields a unique bimodal texture, which can be attributed to the orientation of anisotropic grains during the PHIP process.
(2) (The h-BN grain size has a significant influence on the densification and mechanical properties of Si_3_N_4_/BN composite ceramics. A decrease in h-BN grain size is conducive to densification during sintering. However, the increased formation of the intergranular h-BN phase (< 500 nm) represents a structural flaw, worsening the mechanical properties. Therefore, fine h-BN powders with grain sizes > 500 nm are favorable for the fabrication of densified Si_3_N_4_/BN composite ceramics with beneficial mechanical properties.
(3) The bimodal texture is advantageous in improving thermal conductivity and anisotropy. Under a low sintering temperature of 1800 °C and short dwelling time of 4 h, SNBN2 exhibits a high thermal conductivity of 92.61 W∙m^−1^∙K^−1^ and high thermal anisotropy of 2.17.

Texture control shows unique advantages in improving the overall performance of ceramic materials. The properties of textured Si_3_N_4_/BN composite ceramics could be tailored through careful selection of raw h-BN powder sizes. The newly fabricated textured composite ceramics with reliable overall performance hold great potential in directional heat dissipation of high-power chips.

## Figures and Tables

**Table 1:** A comparison of the properties of the Si_3_N_4_/BN composite ceramics with those of other Si_3_N_4_- and/or BN-based ceramics.

Materials	Fabrication Method	Flexural Strength (MPa)	Fracture Toughness (MPa∙m^1/2^)	Thermal Conductivity (RT, W∙m^−1^∙K^−1^)	Reference
h-BN-MAS	Hot pressing: 1800 °C/30 MPa/1 h	222.9	2.7	94.3	[35]
h-BN-YAG	Hot pressing: 1750 °C/20 MPa/1 h	74.1	3.0	72.8	[36]
β-Si_3_N_4_	Gas pressure sintering: 1525 °C/3 h→1850 °C/3 h	801.0	—	79.42	[8]
β-Si_3_N_4_	Gas pressure sintering: 1900 °C/4 h	857.6	7.7	76	[7]
h-BN/Si_3_N_4_	Hot pressing: 1800 °C/30 MPa/2 h	862.0	10.3	43	[33]
h-BN/Si_3_N_4_	PHIP+GPS: 1700 °C/2 h→1800 °C/4 h	540.6	7.5	92.6	This work

## Data Availability

The original contributions presented in this study are included in the article. Further inquiries can be directed to the corresponding author.
